# Stapokibart, a novel monoclonal antibody targeting the interleukin 4 receptor α subunit: Promising results in a retrospective cohort of patients with bullous pemphigoid

**DOI:** 10.1016/j.jdcr.2026.05.073

**Published:** 2026-07-14

**Authors:** Shufen Wang, Zhuoxue Nong, Dandan Wei, Bingning Yang, Yulin Huang, Xinyu Zhang, Jiaguang Su, Wenjun Zheng, Sijian Wen

**Affiliations:** Department of Dermatology and Venereology, The First Affiliated Hospital of Guangxi Medical University, Nanning, Guangxi, China

**Keywords:** bullous pemphigoid, case series, stapokibart

## Case reports

Bullous pemphigoid (BP), mainly characterized by pruritus and tense blisters, is the most common autoimmune bullous disease, that predominantly affects the elderly population.^1^ Substantial evidence suggests that T helper 2 (Th2) immune response plays an important role in the pathogenesis of BP, marked by the activation of Th2 lymphocytes, elevated immunoglobulin E (IgE) levels, and increased production of type 2 cytokines, such as interleukin (IL) 4 and IL-13.^1^ Among these mechanisms, anti-BP180 IgE binds to Fc epsilon receptor I (FcεRI) on the surface of mast cells and eosinophils, thereby promoting activation of inflammatory cells and triggering an inflammatory cascade, which leads to clinical manifestations such as erythema and blister formation. In addition, the cytokine IL-4 is upregulated in the skin, serum, and blister fluid of patients with BP.^2^^,^^3^ Conventional treatment for BP mainly involves topical corticosteroids and systemic corticosteroids with or without immunosuppressants.^4^ However, this conventional regimen has limited efficacy and can be poorly tolerated by many elderly patients because of significant side effects, particularly in those with multiple comorbidities. In recent years, biologic agents have been explored for the treatment of refractory BP.^5^ The European Academy of Dermatology and Venereology guidelines recommend rituximab (anti-CD20), omalizumab (anti-IgE), and dupilumab (anti–IL-4 receptor α [IL-4Rα] subunit) for patients with recalcitrant BP or contraindications to conventional therapies.^4^ Among these biologic agents, rituximab induces remission of BP by targeting CD20 and depleting autoreactive B cells responsible for autoantibody production. Omalizumab exerts its therapeutic effect by blocking the binding of IgE to FcεRI, thereby reducing mast cell and eosinophil activation. Dupilumab acts further upstream by inhibiting IL-4/IL-13 signaling, modulating the overall Th2 inflammatory axis.^3^ Stapokibart is a novel monoclonal antibody targeting IL-4Rα with an uncommon conformational epitope distinct from dupilumab. Stapokibart exerts potent effects by blocking IL-4Rα–mediated signaling, thereby achieving sustained and thorough inhibition of Th2-type inflammatory responses.^6^ Currently, stapokibart has been approved for the treatment of adults with atopic dermatitis, chronic rhinosinusitis with nasal polyps, and seasonal allergic rhinitis in China.

This case series study included patients with BP who were treated with stapokibart in our department from December 2024 to December 2025. All patients were diagnosed with BP on the basis of medical history; clinical manifestations; serum anti–BP180/230 antibody levels, measured by enzyme-linked immunosorbent assay (Beijing Bolmai Biotechnology Co, Ltd); histopathologic examination; and direct immunofluorescence testing. All patients received subcutaneous injections of stapokibart at an initial dose of 600 mg, followed by 300 mg every 2 weeks. Data on patient demographics, medical history, previous treatments, laboratory test results, and clinical outcomes were collected through review of medical records and follow-up assessments. The Bullous Pemphigoid Disease Area Index (BPDAI) score and levels of anti-BP180 antibody, anti-BP230 antibody, and IgE were analyzed before and after 3 months of stapokibart treatment (or at early discontinuation). All statistical analyses were descriptive and performed using Stata V18.0 (Stata Corp).

We included 10 patients with a confirmed diagnosis of BP, comprising 4 men and 6women, with an mean age of 70.9 years (Table I). Descriptions related to biologic agent induction in patients 1 and 3 have been deleted here. All patients presented with multiple erythematous lesions and tense bullae on the trunk and limbs, with no mucosal involvement observed in any case (Fig 1, patient 3). Prior conventional treatments included topical corticosteroids, systemic corticosteroids, and immunosuppressants (cyclophosphamide and cyclosporine). Specifically, patient 1 had received dupilumab and omalizumab treatment, and patient 5 had received dupilumab. However, these treatments were either ineffective or poorly tolerated.

**Table I:** Demographic and clinical characteristics of 10 patients with BP

BP duration (mo)	Commodities	Prior treatments	Concomitant treatment	BPDAI score∗			ELISA anti-BP180/ELISA anti-BP230 (U/mL)		IgE (IU/mL)		Duration of stapokibart treatment (wk)
				Before	After	ΔBPDAI (%)	Before	After	Before	After	
Case 1, 71 y, F											
16	None	IVMP, dupilumab, omalizumab†, TCS	TCS	67	14	79.1	0.1/0.2	2.3/2.4	324.2	45.1	47 (ongoing)
Case 2, 85 y, F											
1	T2DM	TCS	TCS	88	10	88.6	86.8/140.6	7.9/10.8	184.0	62.1	43 (ongoing)
Case 3, 43 y, M											
1	Hodgkin lymphoma, T2DM, hypertension	IVMP, GS, MTX, TCS	GS (30-5 mg/d)¶, MTX (20 mg/wk), TCS	151	11	92.7	63.0/0.0	9.1/0.0	386.3	55.8	18 (discontinued§)
Case 4, 68 y, M											
14	None	IVMP, GS, CSA, TCS	GS (30-10 mg/d)¶, TCS	74	6	91.9	138.1/121.7	11.2/7.8	619.9	78.2	17 (ongoing)
Case 5, 70 y, F											
9	Herpes zoster, hypertension, colorectal cancer	IVMP, GS, MTX, dupilumab‡	GS (20-10 mg/d)¶, MTX (20 mg/wk), TCS	46	8	82.6	6.2/309.6	1.5/9.5	1500.2	89.9	13 (ongoing)
Case 6, 79 y, F											
2	None	IVMP, GS, TCS	GS (20-5 mg/d)¶, TCS	20	4	80.0	18.0/21.7	2.6/6.8	100.2	38.2	9 (discontinued§)
Case 7, 69 y, M											
2	T2DM, hypertension, hepatitis B, stroke, hyperlipidemia	TCS	TCS	9	1	88.9	23.6/1.5	1.8/0.6	40.8	32.6	6 (discontinued§)
Case 8, 80 y, F											
6	T2DM, hypertension, stroke, atrial fibrillation, Alzheimer disease	IVMP, GS, CSA	GS (30-10 mg/d)¶, CSA, TCS	63	13	79.4	268.28/2.1	57.0/2.3	146.9	52.3	9 (discontinued‖)
Case 9, 60 y, M											
1	None	IVMP, GS, CSA	GS (20-5 mg/d)¶, TCS	51	15	70.6	400.2/8.7	5.7/2.3	4785.6	138.4	6 (discontinued‖)
Case 10, 84 y, F											
3	T2DM	GS, TCS	TCS	68	8	88.2	79.0/120.6	1.6/3.6	7759.2	153.3	44 (ongoing)

*BP*, Bullous pemphigoid; *BPDAI*, Bullous Pemphigoid Disease Area Index; *CSA*, cyclosporin A; *ELISA*, enzyme-linked immunosorbent assay; *F*, female; *GS*, prednisone; *IVMP*, intravenous methyl prednisolone; *M*, male; *MTX*, methotrexate; *TCS*, topical corticosteroids; *T2DM*, type 2 diabetes mellitus；*Δ*, change; *ΔBPDAI (%)*, percentage change in BPDAI score from baseline.

∗ The BPDAI score was evaluated before and after 3 months of stapokibart treatment (or at early discontinuation).

† Patient 1 received dupilumab for 4 months, ending 11 months prior to initiating stapokibart treatment, and omalizumab for 6 months ending 7 months prior to stapokibart treatment.

‡ Patient 5 received dupilumab for 3 months, ending 3 months prior to initiating stapokibart treatment.

§ Patients 3, 6, and 7 discontinued stapokibart treatment because of an excellent clinical response, for which further treatment was deemed unnecessary.

‖ Patients 8 and 9 discontinued stapokibart treatment because of suboptimal response and financial burden.

¶ GS (30-5 mg/d), reduced from a baseline of 30 to 5 mg/d after 12 weeks, with subsequent GS dose adjustments expressed in the same manner.

**Fig 1:**
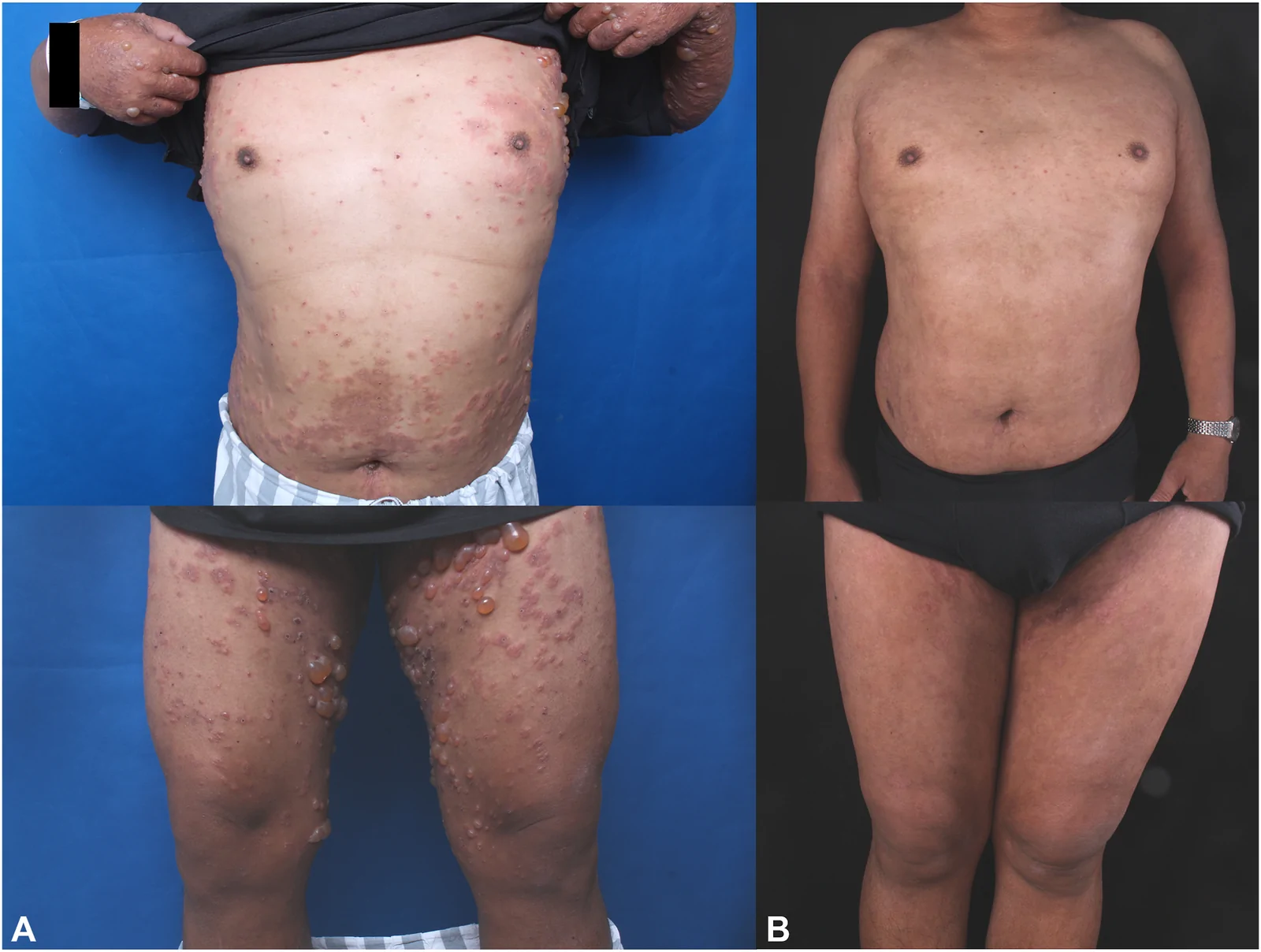
Before stapokibart treatment, patient 3 had multiple erythematous patches and tense blisters and bullae on the whole body. The Bullous Pemphigoid Disease Area Index score was 151 **(A)**. Following 12 weeks of stapokibart treatment, blisters and bullae completely disappeared. The Bullous Pemphigoid Disease Area Index score was 11 **(B)**.

The median duration of stapokibart treatment was 15 (range, 6-47) weeks. At 12 weeks of stapokibart treatment (or early discontinuation), 8 of 10 patients achieved ≥80% reduction in BPDAI. The median BPDAI score decreased from 67.5 (range, 9-151) at baseline to 9, a median absolute reduction of 54.5 points, and a median percentage reduction of approximately 86.7% (Table I). Similarly, median reductions of approximately 94.2% in anti-BP180 and 79.3% in anti-BP230 were observed. Total serum IgE levels decreased to normal in 80% of patients (Table I and Fig 2). In addition, all patients (100%) experienced marked improvement in itching symptoms, with complete resolution observed in 50% of patients. Only 5 patients reported mild or occasional itching at follow-up. Reduction in BPDAI and improvement in itching symptom paralleled declines in anti-BP180/230 antibodies and IgE levels, suggesting a biologically coherent treatment response.

**Fig 2:**
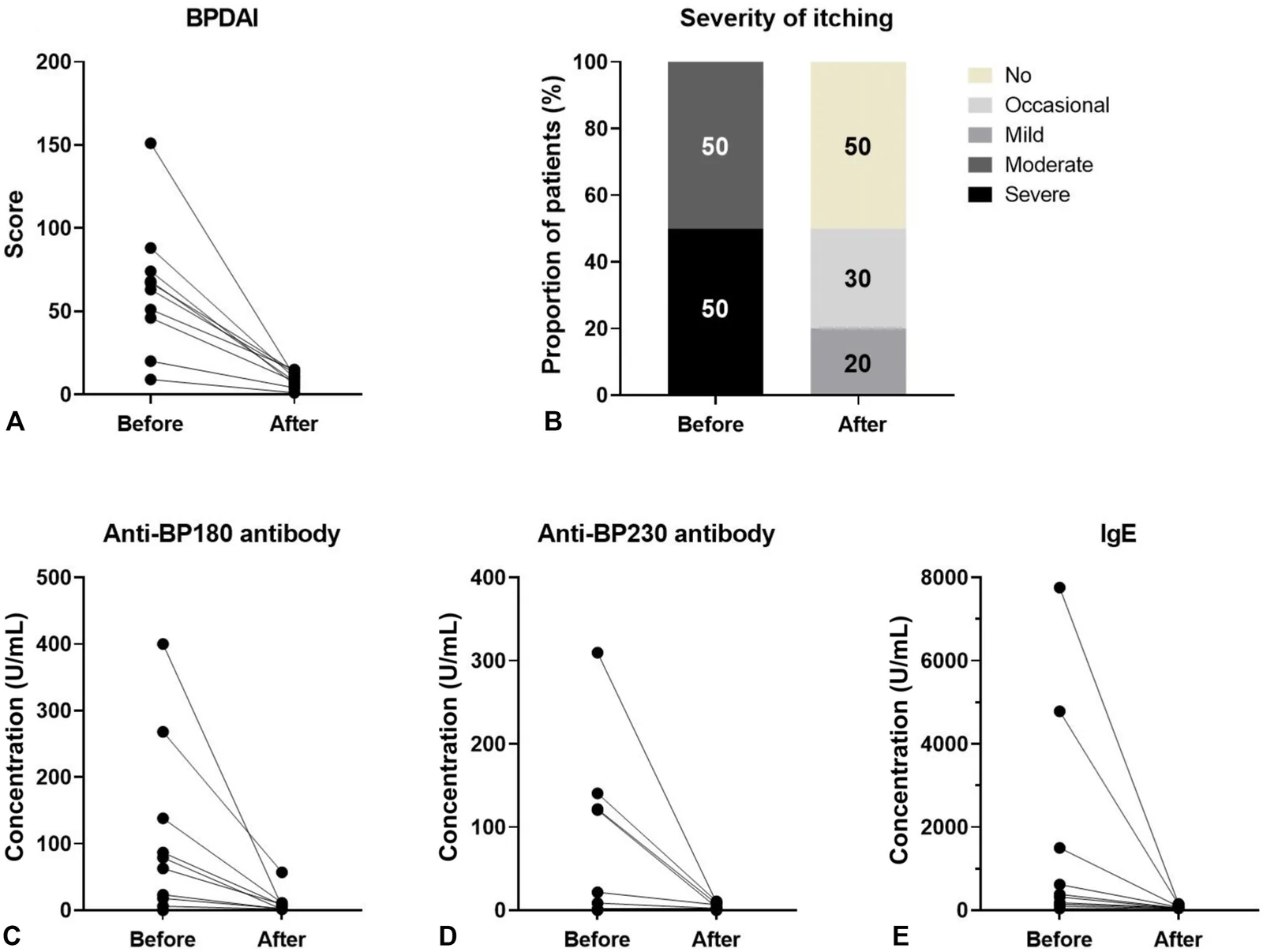
**A,** Bullous Pemphigoid Disease Area Index (BPDAI) score, **(B)** severity of itching, **(C)** anti-BP180 antibody, **(D)** anti-BP230 antibody, and **(E)** IgE levels before and after 3 months of stapokibart treatment (or at early discontinuation). *IgE*, Immunoglobulin E.

Among the 10 patients, 5 remained on stapokibart treatment; 3 patients (patient 3, patient 6, and patient 7) discontinued stapokibart treatment because of an excellent clinical response, for which further treatment was deemed unnecessary; and 2 patients (patient 8 and patient 9) discontinued stapokibart treatment after only 3 and 5 doses because of a poor early response and financial burden. Patients 1 and 5 had received prior treatment with biologic agents; both experienced recurrent new rashes and bullae. However, disease control was achieved in both patients following 12 weeks of stapokibart treatment. Patients 5 and 10 reported occasional new blisters or rashes, respectively, at the follow-up visit at weeks 9 and 6, but these resolved within approximately 1 week of continued treatment. The remaining patients had no relapse during follow-up. Notably, patients were required to receive concomitant low-dose systemic corticosteroids and/or immunosuppressants at the discretion of the treating physician. Therefore, the independent therapeutic contribution of stapokibart cannot be fully determined. However, compared with conventional nonbiologic therapy, the corticosteroid dose was tapered more rapidly, and prednisone was reduced by 5 to 10 mg every 2 weeks, without obvious signs of relapse. None of the patients reported any adverse reactions related to stapokibart treatment.

In the meta-analysis conducted by Cao et al,^3^ the complete remission rates of rituximab, omalizumab, and dupilumab in patients with BP were 70.5%, 67.9%, and 66.7%, respectively, whereas the partial remission rates were 23.8%, 20.8%, and 19.4%, respectively. As a B-cell–depleting agent, rituximab demonstrated the highest response rate in patients with BP; however, it is associated with broad immunosuppression and an increased risk of infection (particularly in elderly patients with BP), and its relapse rate is relatively higher (∼20.5%).^3^ Omalizumab effectively blocks the interaction between IgE and FcεRI. Patients with BP with elevated baseline serum levels of anti–BP180-NC16A IgE achieve higher complete remission rates. However, total serum IgE concentration cannot serve as a predictor of complete remission.^7^ Dupilumab suppresses IL-4/IL-13 signaling upstream and regulates the overall Th2 inflammatory axis; it has been shown to significantly reduce BPDAI scores, alleviate pruritus, rapidly halt new blister formation, and demonstrate a favorable safety profile.^5^ Stapokibart shares the same target as dupilumab (IL-4Rα) but binds closer to the ligand-binding site. In vitro study have shown that it inhibits IL-4/IL-13 signaling, reduces TARC and CD23 expression, suppresses IgE production and histamine release, directly inhibits IgE generation, and exhibits protective effects with good safety in type 2 inflammatory animal models.^6^ A comparative summary of these 4 agents is provided in Supplementary Table I (available via Mendeley at https://data.mendeley.com/datasets/5p9xbr337y/2). Our retrospective study also preliminarily reflects the efficacy and safety of stapokibart in patients with BP.

This study provides preliminary clinical data regarding the use of stapokibart in patients with BP. Substantial reductions in BPDAI scores, pathogenic autoantibodies, and pruritus were observed, without apparent short-term safety concerns. These findings support the biologic rationale of IL-4Rα blockade in BP, particularly given the central role of Th2-driven inflammation in disease pathogenesis.

However, several limitations warrant consideration. The sample size was small, the study was retrospective, and no control group was included. Additionally, patients were heterogeneous in disease duration, severity, and prior treatment exposure. Concomitant corticosteroids and immunosuppressants may have contributed to clinical improvement, and thus, the independent therapeutic effect of stapokibart cannot be fully determined. Furthermore, longer follow-up and controlled studies are needed to better define the optimal positioning of stapokibart within the therapeutic algorithm of BP.

## Conflicts of interest

None disclosed.
